# The Influence of Southwestern Virginia Environmental Conditions on the Potential Ability of *Haemaphysalis longicornis, Amblyomma americanum,* and *Amblyomma maculatum* to Overwinter in the Region

**DOI:** 10.3390/insects12111000

**Published:** 2021-11-06

**Authors:** Amanda Marie Whitlow, Roger Schürch, Donald Mullins, Gillian Eastwood

**Affiliations:** 1 Department of Entomology, Virginia Tech Polytechnic Institute & State University, Blacksburg, VA 24061, USA; amandamw19@vt.edu (A.M.W.); rschurch@vt.edu (R.S.); mullinsd@vt.edu (D.M.); 2 Global Change Center, Virginia Tech Polytechnic Institute & State University, Blacksburg, VA 24061, USA

**Keywords:** ticks, vector, invasive species, overwintering survival, Virginia, Appalachia

## Abstract

**Simple Summary:**

A tick’s ability to survive in cold, harsh winter conditions is influenced by numerous factors including the tick species, the variability in temperature, and the suitability of the overwintering habitat (containing insulation to retain heat). We investigated the influence of elevation and insulation coverage on the survivability of three newly invading ticks, *Amblyomma americanum, Amblyomma maculatum,* and *Haemaphysalis longicornis* and one native tick species already established in southwestern Virginia, *Dermacentor variabilis*. For the invasive species, we found that life stage was the only determining factor in survival for *Haemaphysalis longicornis* and *Amblyomma americanum*, whereas, *Amblyomma maculatum* survival was largely influenced by insulation coverage. *Dermacentor variabilis* survivability was not affected by elevation or insulation coverage in this study.

**Abstract:**

Ticks are susceptible to environmental conditions and, to ensure survival during winter conditions, they adopt a wide variety of physiological and behavioral adaptations including utilization of a suitable niche with insulation (e.g., leaf coverage). To investigate the potential overwintering survival of three tick populations emerging within Appalachian Virginia (*Haemaphysalis longicornis, Amblyomma americanum*, and *Amblyomma maculatum*), both a laboratory experiment assessing super-cooling points and a two-factor (elevation and insulation coverage) field experiment assessing overwintering survivability were conducted across a natural southwestern Virginian winter (2020–2021). *Dermacentor variabilis* adults were included in this study as an example of a well-established species in this region known to overwinter in these conditions. Our study indicated that *A. americanum* and *H. longicornis* wintering tolerance is based on life stage rather than external factors such as insulation (e.g., leaf litter) and elevation. *Amblyomma maculatum* was more likely to survive without insulation. The ability to withstand the extreme temperatures of new regions is a key factor determining the survivability of novel tick species and is useful in assessing the invasion potential of arthropod vectors.

## 1. Introduction

Ticks can be divided into two major families, Ixodidae and Argasidae, commonly referred to as hard and soft ticks, respectively. The majority of ixodid ticks spend most of their life free-living within the environment, only emerging from their microhabitats in the pursuit of a host bloodmeal. Argasid or soft ticks are found in or around sheltered habitats (e.g., burrows and nests) making them dependent on the hosts within and will take multiple bloodmeals during each of their life stages, in comparison to one bloodmeal per life stage in hard ticks. Due to the need for long-term suitable habitat between bloodmeals, hard tick survival is heavily dependent upon the adoption of physiological and behavioral adjustments, specifically during overwintering. Overwintering is the ability of a tick to survive free in the environment while being subject to local climatic conditions and weather during the winter. This includes searching for and obtaining a suitable habitat with an insulating layer (e.g., leaves and stones), entering diapause, or increasing their concentration of cryoprotectants [1]. Insulation coverage is hypothesized to serve as an insulating layer that is pertinent in maintaining relative humidity and temperature to ensure tick survival [2,3,4,5]. It is assumed that variation in climatic differences within microhabitats influences a tick’s ability to overwinter and survive. Controlled studies can elucidate how well ticks are expected to survive conditions within certain regions of the world and how environmental factors influence their survivability. In a study published by Linske et al. [6] investigating the overwintering survival of *I. scapularis* in the northeastern U.S., it was determined that temperatures below −10 °C and a relative humidity below 80% can result in significant tick mortality. The study illustrates the effect of climate conditions on tick survivorship during the winter. *Ixodes scapularis* in particular, has been studied extensively regarding cold tolerance strategies as this species is a significant vector of disease in North America [7,8,9]. Less is known about the overwintering habits of invasive tick species, or of native ticks more traditionally associated with different regions and/or warmer climates. 

While several overwintering studies have been conducted elsewhere in the U.S. [3,6,9], nothing is known about the overwintering of tick populations in mountainous Appalachian Virginia. This region of varying elevation can be subject to harsh winter conditions with snow and high winds. Furthermore, as a belt region between the north and south USA, a variety of tick species persist in Virginia, and three newer tick species of medical or veterinary significance are expanding their populations there. Initial detections of *A. maculatum, A. americanum,* and *H. longicornis* have been made in counties of the western region of the Commonwealth and the New River Valley in recent years [10,11,12], but there is a paucity of knowledge in terms of their ability to tolerate Appalachian winter conditions, and we argue that the existing literature on the climate tolerance of these species does not elucidate how they might survive in this mountainous region of Virginia. 

*Haemaphysalis longicornis* is an invading tick species native to Asia that has been detected in the USA since 2017. Its rapid expansion across 15 states of the USA, including 32 out of 95 counties in Virginia [10], raises concerns about the potential threat to humans, livestock and domestic animals, as this tick species is a significant vector for multiple pathogens elsewhere. While it has yet to be determined whether or not *H. longicornis* poses a significant public health risk in the USA, the tick is a vector for *Theileria orientalis* Ikeda [13], affecting agricultural economies such as that in Virginia, and the species’ current distribution in the states of the eastern seaboard overlaps with several human pathogens of medical and veterinary significance, including *Borrelia* spp., Powassan virus, *Rickettsia* spp., and *Ehrlichia* spp. [14]. While extensive studies have been conducted on the temperature tolerance of *H. longicornis* in Asia, its overwintering survival in the U.S. has yet to be explored. Current climatic predictions of where *H. longicornis* will establish across the U.S. are derived from data from its natural home range [1,15]; local data on how this tick species is surviving winters within the U.S. have not yet been validated.

*Amblyomma americanum* is a known vector of several pathogens (e.g., *Ehrlichia* spp., *Francisella tularensis,* and Heartland virus) and is distributed throughout the eastern U.S. While geographic models of its distribution exist [11,16], the exact distribution of this tick species and the factors limiting its existence within a particular area (e.g., temperature or elevational variation) are not well understood. As *A. americanum* continues to expand its distribution range, attributed to a warming climate and increased availability of hosts, it is important to determine its overwintering ability within southwestern Virginia. Linske et al. [17] determined that the overwintering survivability of *A. americanum* adults emerging in the Northeastern U.S. was dictated by location, but that the presence or absence of insulation (snow and/or leaf litter) had little impact on survival. While this tick species is commonly found in abundance in coastal and Piedmont Virginia, where the ticks overwinter in the nymphal stage, populations in southwestern Virginia have mainly been detected only below 1600 ft (488 m), and the restrictions of elevation and how the species fares during a typical Appalachian winter at higher elevation remains unknown.

*Amblyomma maculatum*, a vector for *Rickettsia parkeri.* and *Hepatozoon americanum* is predominantly found in the southern U.S. (e.g., coastal areas bordering the Gulf of Mexico and the Atlantic Ocean). However, *A. maculatum* has been identified along the eastern coast of Virginia, as well as sporadically within the southwestern region, North Carolina, Delaware, and Kentucky [12]. Incidental reporting of *A. maculatum* further north is generally attributed to expanding white-tailed deer populations and migratory birds [15]. With the expansion of reservoir host populations and the influence of migratory birds, the introduction of such ticks into new regions poses significant health risks. It is pertinent to investigate how *A. maculatum* could fare with the climatic conditions in these new areas, such as southwestern Virginia, and assess the likelihood of the species becoming established.

In the experiments described here, only adult females of *H. longicornis* were used, since only the parthenogenetic strain (reproducing without mating) with no males has been found within the U.S. For each of the other tick species, both adult males and females were used, held in separate vials to avoid potential mating. Nymph and larval life stages were included to assess the influence of life stage on survival, as different tick species have different seasonal peaks. *Dermacentor variabilis* is able to overwinter in any of its post-egg life stages [18]. However, the life stage in which *A. maculatum* overwinters varies depending upon geographical location and is more challenging to track [19]. *Haemaphysalis longicornis* development and subsequently the overwintering life stage is dictated by temperature, host availability, day length, and humidity [20].

While each of these species have been documented within the region, it is not known how well they survive over the winter and whether or not southwestern Virginia is suitable for their populations. Virginia is a large commonwealth with a diverse geology, and it is pertinent to note the potential influence of climate change within the area. Although the state may be considered to have a milder climate than Northeastern or Midwestern U.S., the Appalachian Mountains along the west of the state present their own elevational macrohabitat, with harsh winter conditions arising. With new tick species such as *H. longicornis* recently reported, and tick populations of vectors such as *A. americanum* and *A. maculatum* expanding into the region, understanding the capacity of these ticks to overwinter within southwestern Virginia will provide useful information in assessing vector range expansion, establishment, and public health risk. 

## 2. Materials and Methods

The potential for different tick species to survive in southwestern Virginia winters was assessed in two ways. Firstly, a field experiment was conducted holding tick specimens at various life stages at study sites of differing elevations in the region over a natural typical winter. Secondly, laboratory analysis of freezing survival limits was determined by cooling the ticks at a specific rate until detection of their super-cooling temperatures. 

### 2.1. Overwintering Study

A field study was conducted during the winter of 2020–2021 to assess the overwintering survival ability of *H. longicornis, A. maculatum, A. americanum,* and *D. variabilis* in a typical window of southwestern Virginia winter conditions. Adult *D. variabilis* were included within this study to serve as a control, since this is a local tick species that is well established within the study region. Different elevations are expected to produce different climatic forces, and leaves can serve as an insulating layer and potentially aid the survival of ticks. Thus, a two-factor design was used to examine the effect of (i) elevation and (ii) insulating ground coverage on overwintering survival during this study, based upon a modified field protocol established by Linske et al. [6,17]. A total of 24 (4-L) pots containing tick specimens (details below) were placed in the ground between the months of October 2020 (before the first frost) and April 2021 (after the last frost). For the first treatment factor in this design (elevation), 8 pots were placed at each of three different elevations in the study region: 1000 ft/305 m (Roanoke County), 2000 ft/610 m (Montgomery County), and 3000 ft/914 m (Floyd County). For the second factor (ground coverage), at each elevation where the 8 pots were placed, four of the pots were subjected to leaf removal while the other four did not have leaves removed (Supplementary Figure S1); thus, there were 4 replicates of each condition (elevation × ground coverage). A HOBO logger (Onset Comp, Bourne, MA, USA) was placed at each of the three elevational site locations to monitor the temperature and relative humidity of the general site (with a reading every 6 h).

The pots were approximately 20 cm deep and backfilled with the soil that had been removed from the pots’ site up to the top inch (Supplementary Figures S2 and S3). The pots were placed randomly at each site in similar conditions (e.g., habitat and canopy cover). Holes had been cut into the lid top and the bottom of the pot, covered in mesh to ensure containment of the ticks. Each pot contained a total of nine (9) tick vials containing the unfed larva, nymph, and adult of each tick species. *Amblyomma maculatum* ticks used in this study were obtained from Oklahoma State University, and all other tick species were provided by BEI Resources (CDC, Atlanta, GA, USA). Ticks were held in a 5 mL plastic vial with mesh-covered holes in the top and bottom to promote air flow (and without any material such as cotton balls in the vials). The holes were covered in mesh to prevent ticks from escaping. There was a different vial per tick species group. Nymph vials contained 3–4 nymphal ticks, adult vials contained 1 adult tick, larval vials contained 15 larval ticks. A Thermochron iButton (iButtonLink, LLC, Whitewater, WI, USA) was placed within each pot to monitor the temperatures experienced by the ticks within the pots. Once the tick vials were inside the pot’s soil, the pot was sealed with a lid (also with mesh-covered air holes). Protective chicken wire was pinned over each pot to limit the possibility of wildlife tampering with the pot. No ticks were able to escape, and individual ticks used in the field experiment were counted out and counted back in. Pots were checked bi-weekly over the winter, to ensure they had not been disturbed and that appropriate leaf coverage remained. At the end of the winter, after the last frost in the following spring, the field experiment terminated. Pots were retrieved from the environment, and tick specimens were examined to assess survivorship. All ticks that were placed within the vials at the beginning of the winter were retrieved and accounted for (both dead and alive; Table 1).

### 2.2. Statistical Analysis

#### 2.2.1. Overwintering Assessment

We used the rethinking package in R (version 3.6.3) for analysis of the survival outcomes within a Bayesian framework [21]. We considered survival as a binomial outcome for each species separately, and we modeled the probability of survival as a two-way interaction of our treatment (no leaves vs. leaves) and elevation (the equivalent of 1000, 2000, and 3000 ft). In addition, we adjusted these target models for either life stage (*A. americanum* and *H. longicornis*) or sex (*D. variabilis*). In *A. americanum* we pooled the adult sexes because the sex was only available for adults. Only nymphs of unknown sex were tested in *A. maculatum* and consequently we did not adjust for life stage or sex in this species. In all models we allowed each pot (our blocking factor) to have its own intercept for survival. Priors for the linear parameters (intercept and predictors) were generally set to a mean of 0 with a standard deviation of 10. The intercept for individual boxes was set at a mean of 0 with the prior for the hyperparameter σ_box_ set as a half-Cauchy distribution with mean μ = 0 and the scale parameter σ = 1 (more details on the model parameterization can be gleaned from the code). Each model was run with 4 chains and 8000 samples per chain. The first 4000 samples were used for warm-up before sampling from the second 4000 samples. Since this was a confirmatory experiment, we did not perform model selection. Instead, we determined the odds ratio (a measure of the association between a treatment and its outcome), with 95% credible intervals, of survival for the subgroup’s relative survival at 1000 ft and no leaves, and we present the figures for predicted survival (95% prediction intervals) based on the posterior samples from these models. These latter posterior predictions were performed for blocks with an average intercept rather than for an unknown pot [22]. 

In a second step, and because the litter treatment was unlikely to have different effects at different altitudes in any of our species, we fitted models without the interaction term but still adjusting for life stage or sex as described above. The main effect presented for life stage or sex is taken from these reduced models. We provide maximum a posteriori (MAP) *p*-values derived from the posterior as described by Kelter [23].

#### 2.2.2. Super-Cooling Point Assessment

As a second evaluation of potential winter survival, the ability of tick species to withstand certain limits of super-cooling temperatures was experimentally determined for each tick species and life stage.

A 10 L refrigerated circulating water bath (Isotemp Digital Refrigerated/Heated Water Circulator, Thermo Fisher Scientific Inc., Waltham, MA, USA) attached to a Stir-Kool Model SK-12 (Thermoelectrics Unlimited Inc., Wilmington, DE, USA) was used to lower temperatures to determine the super-cooling points of *H. longicornis, D. variabilis, A. maculatum,* and *A. americanum.* Temperatures were calculated using Omega thermocouples (Omega Engineering, Stamford, CT, USA) arranged in an arena of five thermocouples [24]. Ticks were placed on their dorsal side and adhered to the thermocouples using thermal grease. Ticks were cooled at a rate of 0.03 °C per minute. To maintain temperatures, the stacking order of the cooling arena placed on the Stir-Kool plate was as follows: 1–5 mm thick aluminum square, four 2 mm thick pieces of compressed corrugated cardboard, tick arena, wax paper, foam insulation, and small aluminum weight. An insulating arena was constructed from layers of polypropylene thermally insulating sheets and duct tape to encompass the ticks and stacking order. Sixteen adults and 35 nymphs of each species were used to collect these data. Average super-cooling temperatures were then calculated for each life stage, tick species, and gender if applicable. Super-cooling temperatures were appraised by trimming the raw data generated by the Omega thermocouples to detect heat spikes. The super-cooling temperature is the lowest point prior to the spike in temperature; when the tick reaches the super-cooling point, heat is released and the tick freezes. A Student’s t-test was conducted to determine whether life stage within species was significant for the temperature at which the ticks froze.

## 3. Results

### 3.1. Overwintering Results 

#### 3.1.1. Survival of *A. americanum* Does Not Depend on Leaf Litter or Elevation but Depends on Life Stage

**Figure 1:**
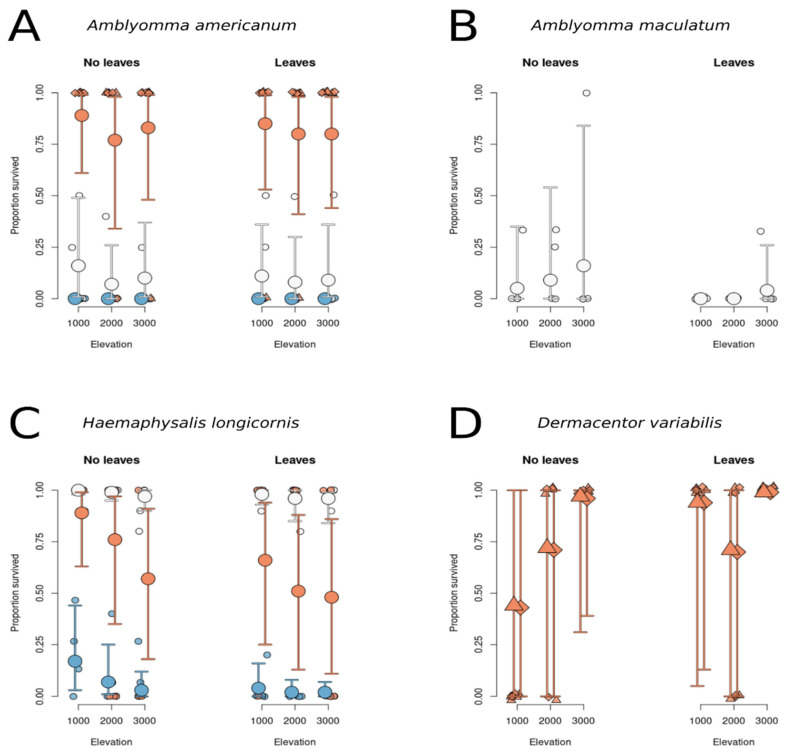
Raw survival data and predicted survival (+/− 95% prediction interval) of adult (orange), nymphal (white), larval (blue) ticks for (**A**) *Amblyomma americanum*, (**B**) *Amblyomma maculatum*, (**C**) *Haemaphysalis longicornis*, and (**D**) *Dermacentor variabilis*. In (**D**), diamonds are males, triangles denote females.

In the light of our models, it is unlikely that in our experiments the survival of *A. americanum* depended on an interaction between leaf litter and elevation (odds ratios: leaves vs. no leaves for 2000 vs. 1000 ft: 1.9 (0.0 to 114.9), *p* = 0.91; leaves vs. no leaves for 3000 vs. 1000 ft: 1.5 (0.0 to 81.3), *p* = 0.96). Models with the interaction term removed did not indicate that leaf litter (leaves vs. no leaves: 0.8 (0.2 to 3.9), *p* = 1) or elevation (2000 vs. 1000 ft.: 0.8 (0.2 to 3.9), *p* = 0.75; 3000 vs. 1000 ft.: 0.8 (0.2 to 3.9), *p* = 0.82) affected survival on their own. However, life stage was a likely predictor of survival, with nymphs and adults having relative odds ratios compared to larva of 1564 (37.2 to 1,114,883) (*p* = 0) and 150,369 (2503 to 121,407,803) (*p* = 0), respectively. These high odds ratios are primarily a reflection of the zero survival at all elevations and treatment levels for larva, whereas nymphs had an overall survival rate of 7% (1 to 17%) (difference: 7% (1 to 17%), and adults a survival rate of 83% (64 to 95%) (difference: 83% (64 between leaf litter and elevation) (odds ratios: leaves vs. no leaves for 2000 vs. 1000 ft: 0.0 (0.0 to 11,223), *p* = 0.9; leaves vs. no leaves for 3000 vs. 1000 ft: 94.8 (0.0 to 15,372,267), *p* = 0.78), nor on elevation (see Figure 1, odds ratios: 2.4 (0.0 to 1756), *p* = 0.94; 14.4 (0.0 to 19,446), *p* = 0.58), and overall, we did not find an indication that survival of the nymphs might be diminished in leaf litter (odds ratio: 0.0 (0.0 to 1.6), (*p* = 0.34), even though survival differed considerably between the two treatments (mean survival probabilities for no leaves: 8% (0 to 37%); leaves: 0% (0 to 2%); difference: −8.0% (−36.5 to −0.0%)).

#### 3.1.2. Survival of *H. longicornis* Does Not Depend on Leaf Insulation or Elevation but Depends on Life stage

The survival of *H. longicornis* did not depend on an interaction between leaf litter and elevation (odds ratios: leaves vs. no leaves for 2000 vs. 1000 ft: 1.5 (0.1 to 45.5), *p* = 0.97; leaves vs. no leaves for 3000 vs. 1000 ft: 3.3 (0.1 to 101.2), *p* = 0.77). Once we removed the interaction term from the models, we did not find that leaf litter (leaves vs. no leaves: 0.3 (0.1 to 1.3), *p* = 0.23) or elevation (2000 vs. 1000 ft.: 0.4 (0.1 to 2.1), *p* = 0.48; 3000 vs. 1000 ft.: 0.2 (0.0 to 1.1), *p* = 0.17) affected survival on their own. However, life stage was a likely predictor of survival, with nymphs and adults having relative odds compared to larvae of 2471 (683 to 11,308) (*p* = 0) and 70.5 (20.2 to 277) (*p* = 0), respectively. Whereas larva had an overall survival rate of 2% (1 to 6%), nymphs had an overall survival rate of 98% (95 to 100%) (difference: 95% (92 to 98%)), and adults a survival rate of 59% (28 to 85%) (difference: 57% (27 to 81%)).

#### 3.1.3. Survival of *D. variabilis* Is Not Predicted by Elevation or Leaf Litter Treatment

We did not find an effect of both elevation and treatment on the survival of adult *D. variabilis* (odds ratios: leaves vs. no leaves for 2000 vs. 1000 ft: 0.0 (0.0 to 102,641), *p* = 0.58; leaves vs. no leaves for 3000 vs. 1000 ft: 20.9 (0.0 to 1,550,134,946), *p* = 0.97), nor did survival depend on leaf litter alone (leaves vs. no leaves: 1517.0 (0.0 to 3,593,852,626), *p* = 0.52) or elevation alone (2000 vs. 1000 ft.: 0.9 (0.0 to 1,906,412), *p* = 1; 3000 vs. 1000 ft.: 188,390 (0.0 to 1,229,880,296,090), *p* = 0.28) in our experiments (see Figure 1). The sexes did not differ in their survival, with females being equally likely to survive as males (odds ratios: 1.4 (0.0 to 9627.7), *p* = 1).

### 3.2. Temperatures, Relative Humidity, and Precipitation Experienced at Each Elevation

Average, minimum, and maximum temperatures and relative humidity values at each elevation were calculated from the HOBO logger placed at each site (Table 2). From data collected by the NOAA (National Oceanic and Atmospheric Administration) [25], our elevational sites experienced similar amounts of precipitation (around 25 inches) during the study period, implying that there was no significant difference in precipitation amounts between the sites. Snow has been known to provide insulation [6]; however, snow retained for longer than two days is limited within the region and was not studied. Temperatures logged by the iButtons within each pot were the same across treatments (leaves vs. no leaves). 

### 3.3. Super-Cooling Point Results

Average super-cooling point temperatures, standard deviations (SD), and standard errors (SEM) were calculated from the individual super-cooling temperatures of each tick species by life stage (Table 3). *A. americanum* adult males had statistically lower super-cooling temperatures when compared to adult females (*p* = 0.01). *Amblyomma americanum* nymph super-cooling point temperatures were statistically lower than those of the adults of the same species (*p* < 0.0001). *Haemaaphysalis longicornis* nymphs had statistically lower super-cooling point temperatures than the adults (*p* = 0.03). There was no statistical difference between *D. variabilis* adult male and adult female super-cooling temperatures (*p* = 0.07). Only *A. maculatum* nymphs were assessed for super-cooling point temperatures due to the availability of this tick species.

## 4. Discussion

Ticks spend the majority of their lives living in an environment where they are subjected to the whim of climatic conditions. A major threat for overwintering arthropods such as ticks is the lower temperatures that occur during midwinter. We therefore considered how three invading tick species in Appalachian Virginia might withstand the coldest natural conditions experienced in the region at different elevations, as well as the ability of each species to tolerate super-cooling. Our results showed that three emerging tick species in southwestern Virginia, *H. longicornis, A. americanum,* and *A. maculatum,* as well as the native tick species *D. variabilis*, had varying survival rates, and that leaf insulation and elevation are not necessarily crucial determinants of survival. Virginian *A. americanum* survival, for example, only depended upon the life stage they had reached (adults faring best), rather than external factors such as elevation and insulation (leaf coverage). These results corroborate a previous study conducted on this tick species in the Northeastern US that investigated the influence of snow and leaf coverage, confirming insulation has no effect on overwinter survival [17]. In contrast, the survival of invasive *A. maculatum* is likely to be influenced by the presence of leaf litter, survival being higher without the presence of leaves. Insulation has been determined to be a critical factor in the overwintering survival for certain tick species in the Northeastern U.S., (i.e., for *I. scapularis*) [6], whereas for others it appears to have less of an influence [17]. 

While we did not find any significant interaction between the survival of *A. maculatum* and elevation, insulation is likely to contribute to its overwintering survival. Although in this study we did not find evidence to suggest that survival diminished with insulation (leaves), survivability was higher in *A. maculatum* subjected to no insulation. This may be attributed to their xerophilic nature (preference for drier conditions) [15]. We only assessed the nymphal life stage (the common life stage that the tick overwinters in) due to a lack of availability of tick specimens; thus, future research could investigate how other *A. maculatum* life stages fare during a Virginian winter when compared to other regions where this tick is commonly found [19]. 

*Haemaphysalis longicornis* survival was also dependent upon the life stage in which the tick was overwintering. Both nymph and adult *H. longicornis* had comparable survival rates, indicating that ticks in these life stages are equally capable of surviving over the winter. *H. longicornis* poses a significant threat to animal health (i.e., vectoring *T. orientalis* Ikeda to cattle), and therefore by identifying the influencing factors affecting how this species survives, pest management programs can utilize this knowledge in developing future protocols [26]. 

In contrast to *H. longicornis, A. americanum,* and *A. maculatum,* the tick included as a native control in the field study, *D.variabilis*, is established throughout the eastern U.S., along seaboard states from the Gulf of Mexico to New England, as well as in parts of Canada [27]. With expanding populations of *Amblyomma* spp. (*A. americanum* and *A. maculatum*) throughout the eastern U.S., it was pertinent to address concerns regarding their overlap with *D. variabilis,* which is commonly found throughout southwestern Virginia [11,12,16,28]. *Dermacentor variabilis,* a vector for *R. rickettsii* and *Francisella tularensis,* is a desiccation-tolerant tick found along forest edges, roadways, and trails. *Amblyomma* spp. have a waxy cuticle that prevents water loss and allows them to survive adverse climatic conditions (e.g., hotter temperatures), and they are commonly found in dense underbrush. A recent study suggested that factors such as habitat and vegetation are not enough to determine the population distributions of *D. variabilis, A. americanum,* and *A. maculatum* [29].

There are more studies of the overwintering capabilities of *D. variabilis* than of the other invasive tick species [5,16,18,30]. Insulation and snow cover were proven to be significant factors in terms of mortality, as *D. variabilis* is subject to desiccation [5]. *Dermacentor variabilis* also overwinters in any of its post-egg life stages [18]. In our study, we found no significant influence of insulation on overall survival of adult *D. variabilis*, which differs from a previous study [5]. This may be attributed to the location in which the study was conducted (e.g., Massachusetts vs. Virginia) with certain regions experiencing different winter conditions (temperature, snowfall, etc.). Additionally, continuous changes in climatic conditions, for example, increases in annual global temperatures [31] lead to milder winters enabling ticks to survive with or without insulation. We placed individual Thermochron iButtons within each pot to monitor temperatures for each of the different insulation treatments. There was no significant difference between temperatures in pots with leaves compared to those without, suggesting that other effects from the leaves influence survival (e.g., light, increased moisture, draft, etc.). 

With expanding ranges in tick distribution and the introduction of invasive species, it is pertinent to assess how they may survive in novel areas including different elevations. The influence of elevation on tick overwintering survival has not previously been examined in other studies of cold hardiness. Virginia is divided into five different climate regions as a result of global weather patterns and the diverse geological landscape of the state [32,33], and here, as we focused on southwestern Virginia (Appalachia to Blue Ridge) which experiences the most severe winter conditions, we encompassed sites with a range of elevations. While one may have expected temperatures to drop with increasing elevation, our middle elevation site in fact experienced the coldest average temperatures; thus, the association between survival and elevation could be complex and should be revisited in another study. Furthermore, we acknowledge that the field study only considered a single snapshot (2020–2021) of the winter conditions in the region, and therefore, despite that period presenting a fairly harsh typical winter, further multi-year studies could examine survival under field conditions more extensively.

It is important to acknowledge studies investigating the overwintering abilities of ticks in other parts of the world (e.g., Europe) and how they aid in our understanding of tick survival. Similarly to ticks found in the U.S., tick species (e.g., *Ixodes ricinus*) in Europe are also expanding their geographical distribution. The distribution shifts are mainly attributed to changes in climatic conditions, anthropogenically induced changes, distribution and availability of hosts, habitat management, etc. [34,35]. With a warming climate, ticks are likely to expand their distribution and therefore present a threat to human safety, as supported by the study investigating the overwintering ability of *Dermacentor reticulatus* [36]. It was found that *D. reticulatus* was able to overwinter well in a temperate climate (defined by moderate rainfall throughout the year, mild summers, and cool winters similar to those of southwestern Virginia) [36]. Thus, as ticks continue to emerge in new, previously undocumented regions in the U.S., it is crucial to understand the factors limiting their establishment success. 

The cold tolerance of tick vectors may be pertinent for pathogen infection. In other studies, it has been shown that *Anaplasma phagocytophilum* infection of *I. scapularis* has been linked to increased cold tolerance and overall survivability of *I. scapularis*; this is attributed to *A. phagocytophilum* inducing the expression of the antifreeze glycoprotein, *iafgp* [37]. Therefore, (although not investigated here) it is pertinent to note that infected vectors may have a slight advantage in survivability in comparison to uninfected vectors. If other pathogens confer the same advantage to the tick vector in terms of surviving the winter, then the risk of acquiring a tick-borne disease is likely to drastically increase. Due to time constraints and biosecurity limitations when conducting field studies (e.g., placing infected ticks into the environment), only uninfected tick vectors (*H. longicornis, A. americanum,* and *A. maculatum*) were assessed for their overwintering abilities. However, it is important to recognize that there are other factors, such as pathogen infection or life stage, that may influence overwinter survival, in addition to temperature, relative humidity, and insulating factors (e.g., leaf or snow coverage).

During our laboratory assessment of cold hardiness by determining super-cooling points, nymphal-stage ticks (*A. americanum* and *H. longicornis*) were found to be more cold-hardy, with lower super-cooling points, when compared to adult ticks. Nymphal *A. americanum* and *H. longicornis* tolerance for colder temperatures suggests a potential to overwinter better than the other active life stages. When combining the results from the field and laboratory studies, *A. americanum* adults in the field had a higher survival rate than nymphs. This contrast may indicate that while nymphs are able to withstand colder temperatures in a controlled laboratory setting, ticks’ overwintering survivability is dictated by how well they are able to acclimate to fluctuating temperatures and relative humidity in the field [3]. *Amblyomma maculatum* nymphs showed similar average super-cooling temperatures to *H. longicornis* and *A. americanum* nymphs. Due to specimen availability, and the fact that nymphs are the stage at which this species is suggested to overwinter, only *A. maculatum* nymphs were assessed for their cold hardiness. This suggests that *A. maculatum* nymphs would have similar survival rates to those of *H. longicornis* and *A. americanum.* However, the low overwintering survival rates of *A. maculatum* in the field study suggest that Appalachian Virginia may not be suitable to sustain invasions of this tick species. 

## 5. Conclusions

In conclusion, our study found that overall elevation and insulation coverage had limited effect on the predicted overwintering survival of *A. americanum, H. longicornis,* and *A. maculatum* in southwestern Virginia. However, aside from these ambiental factors, the key factor in survival for *A. americanum* and *H. longicornis* was the life stage of the tick. We therefore suggest that other factors, and maybe only the opportunity for incursion alone, would limit the presence of these novel tick species in this central Appalachian region. Understanding the cold tolerance of tick vectors is crucial for predicting their activity and future distribution ranges. By recognizing influencing factors on tick overwintering survival (e.g., insulation coverage, elevation, temperature, relative humidity, life stage, gender, etc.), expansion and health risks associated with ticks can be more accurately predicted.

## Figures and Tables

**Table 1:** Details of ticks used within the field experiment: number of each species and replicates placed at different elevations and insulation treatments, and how many survived. Note, only nymphs were used for *Amblyomma maculatum*.

	No Leaves, # of Replicates	No Leaves, Survived/Placed	Leaves, # of Replicates	Leaves, Survived/Placed
***Amblyomma americanum***	48	26/249	48	25/253
**1000 ft/305 m**	16	10/80	16	9/86
Larva	4	0/56	4	0/60
Nymph	4	3/16	4	3/18
Adult (males)	4	4/4	4	4/4
Adult (females)	4	3/4	4	2/4
**2000 ft/610 m**	16	8/86	16	8/84
Larva	4	0/60	4	0/60
Nymph	4	2/18	4	2/16
Adult (males)	4	3/4	4	4/4
Adult (females)	4	3/4	4	2/4
**3000 ft/914 m**	16	8/83	16	8/83
Larva	4	0/60	4	0/60
Nymph	4	1/15	4	2/15
Adult (males)	4	4/4	4	2/4
Adult (females)	4	3/4	4	4/4
***Amblyomma maculatum***	12	6/39	12	1/38
**1000 ft/305 m**	4	1/13	4	0/13
**2000 ft/610 m**	4	2/13	4	0/13
**3000 ft/914 m**	4	3/13	4	1/12
***Haemaphysalis longicornis***	36	147/312	36	127/311
**1000 ft/305 m**	12	56/104	12	44/103
Larva	4	13/60	4	3/60
Nymph	4	39/40	4	38/39
Adult	4	4/4	4	3/4
**2000 ft/610 m**	12	48/104	12	42/104
Larva	4	7/60	4	0/60
Nymph	4	40/40	4	38/40
Adult	4	1/4	4	4/4
**3000 ft/914 m**	12	43/104	12	41/104
Larva	4	5/60	4	0/60
Nymph	4	37/40	4	39/40
Adult	4	1/4	4	2/4
***Dermacentor variabilis***	24	14/24	24	20/24
**1000 ft/305 m**	8	0/8	8	8/8
Adult (males)	4	0/4	4	4/4
Adult (females)	4	0/4	4	4/4
**2000 ft/610 m**	8	6/8	8	4/8
Adult (males)	4	3/4	4	2/4
Adult (females)	4	3/4	4	2/4
**3000 ft/914 m**	8	8/8	8	8/8
Adult (males)	4	4/4	4	4/4
Adult (females)	4	4/4	4	4/4

**Table 2:** Average, minimum, and maximum temperatures and relative humidity (RH) values at each elevational site.

Elevation	Minimum Temp (°C)	Average Temperature (°C)	Maximum Temperature (°C)	Minimum RH	Average RH	Maximum RH
1000 ft/305 m (Roanoke)	−8.66	6.33	24.97	18.458	81.76	100
2000 ft/610 m (Montgomery)	−5.51	5.60	20.34	1.00	89.95	100
3000 ft/914 m (Floyd)	−6.96	5.97	26.09	19.44	88.85	100

**Table 3:** Average super-cooling point temperatures, standard deviations, and standard errors for each tick species by life stage.

Tick Species/Life Stage	Average Super-Cooling Point (°C)	Standard Deviation (SD)	Standard Error (SEM)
*Amblyomma americanum* Nymph	−23.53	1.56	0.29
*Amblyomma americanum* Adult Male	−19.74	1.62	0.42
*Amblyomma americanum* Adult Female	−17.30	4.38	1.13
*Haemaphysalis longicornis* Nymph	−24.08	1.40	0.50
*Haemaphysalis longicornis* Adult Female	−22.64	1.44	0.38
*Dermacentor variabilis* Adult Male	−16.04	7.21	1.86
*Dermacentor variabilis* Adult Female	−20.16	4.91	1.23
*Amblyomma maculatum* Nymph	−22.23	2.42	0.43

## Data Availability

Data are contained within the article or supplementary material.

## Supplementary Materials

The following are available online at https://www.mdpi.com/article/10.3390/insects12111000/s1. Figure S1. Overwintering field experimental design for ticks held from October 2020–April 2021 addressing factors of elevation and insulation. A total of 24 overwintering containers were placed with 8 per elevation and 4 per treatment of insulation coverage (leaf removal vs. no leaf removal). Figure S2. Composition of overwintering tick containment units. Figure S3. Placement of tick containment vials (*n* = 9) in overwintering containers (*n* = 24); (a) with lid off, (b) with lid in place. Ticks were placed within the soil, 3 cm deep.
