# Supplementary figures and images for: The Influence of Southwestern Virginia Environmental Conditions on the Potential Ability of Haemaphysalis longicornis, Amblyomma americanum, and Amblyomma maculatum to Overwinter in the Region
Source: Insects. 2021 Nov 6;12(11):1000. doi: 10.3390/insects12111000 (PMC8622198; doi:10.3390/insects12111000)

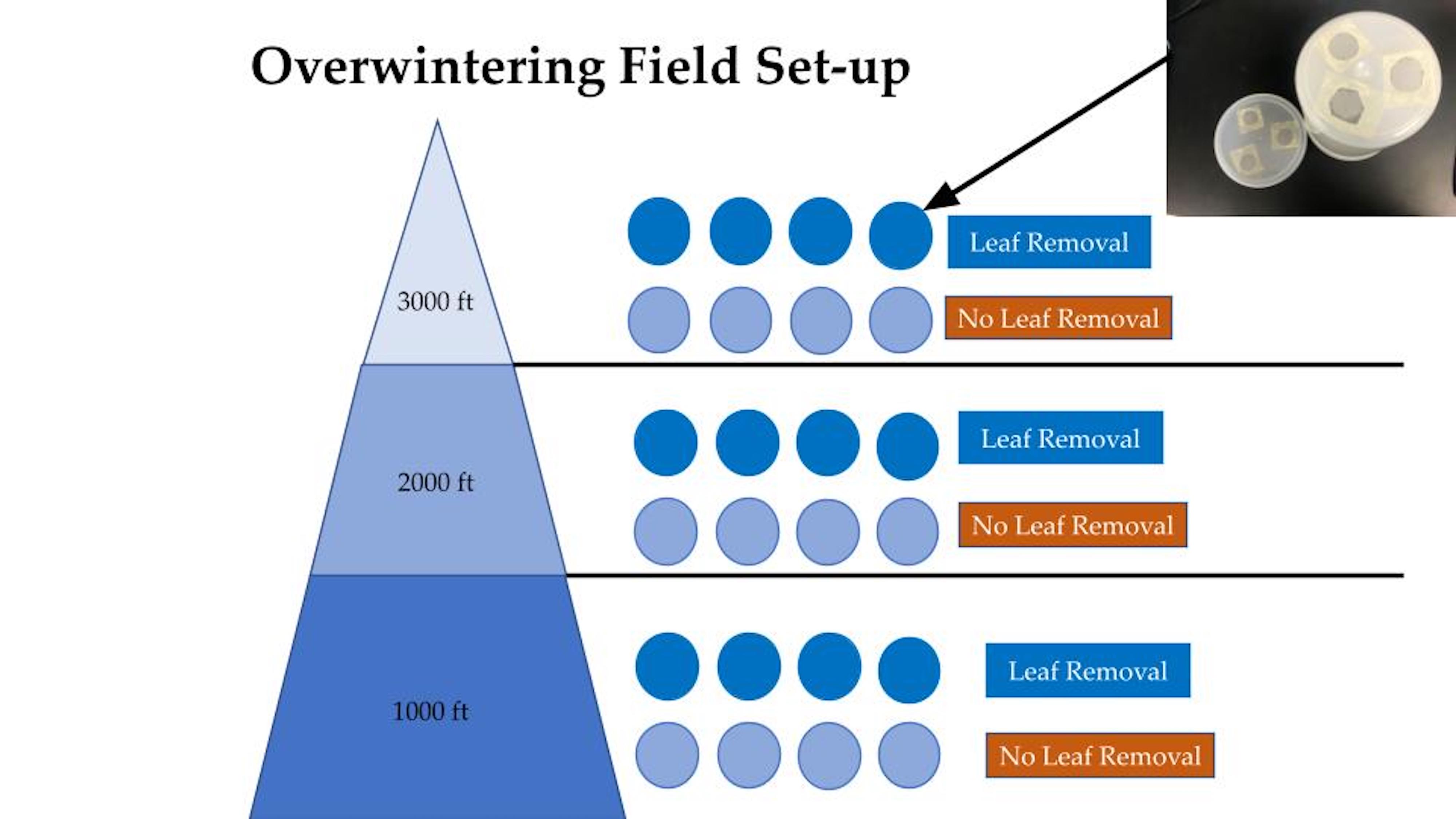

Supplement: Supplementary file 1 [file insects-12-01000-s001.zip › Figure S1.jpg]

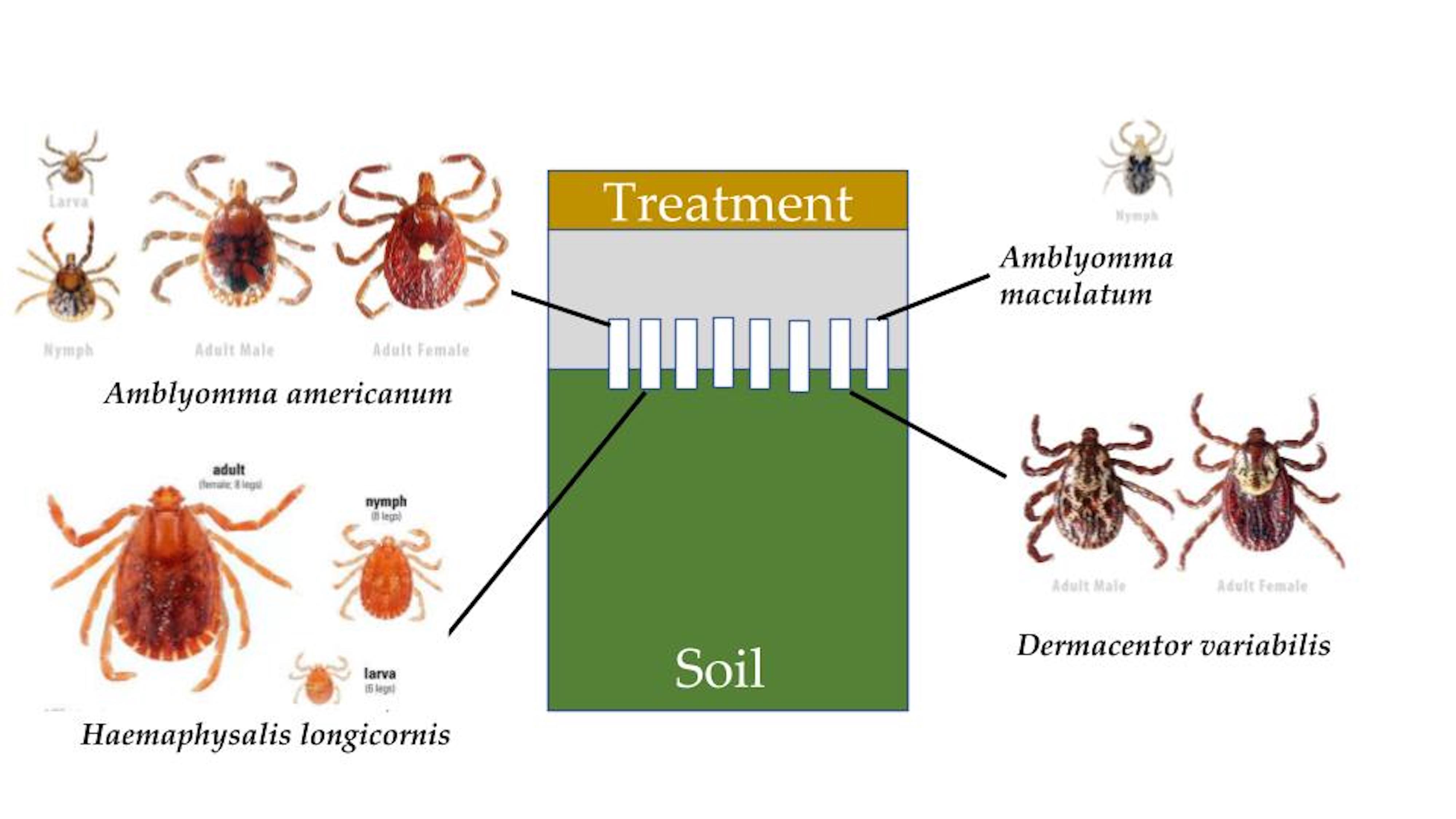

Supplement: Supplementary file 1 [file insects-12-01000-s001.zip › Figure S2.jpg]

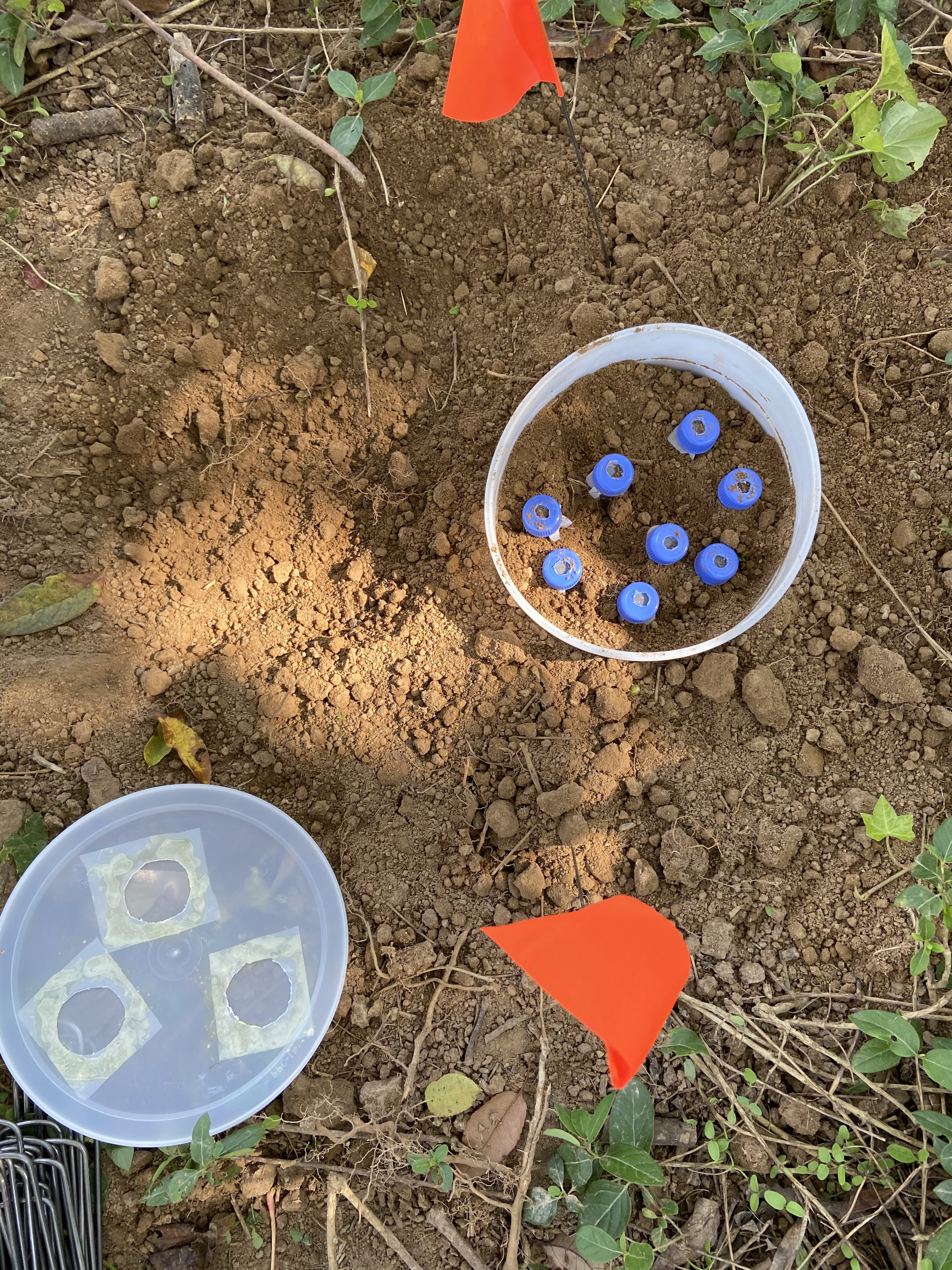

Supplement: Supplementary file 1 [file insects-12-01000-s001.zip › Figure S3-a.jpg]

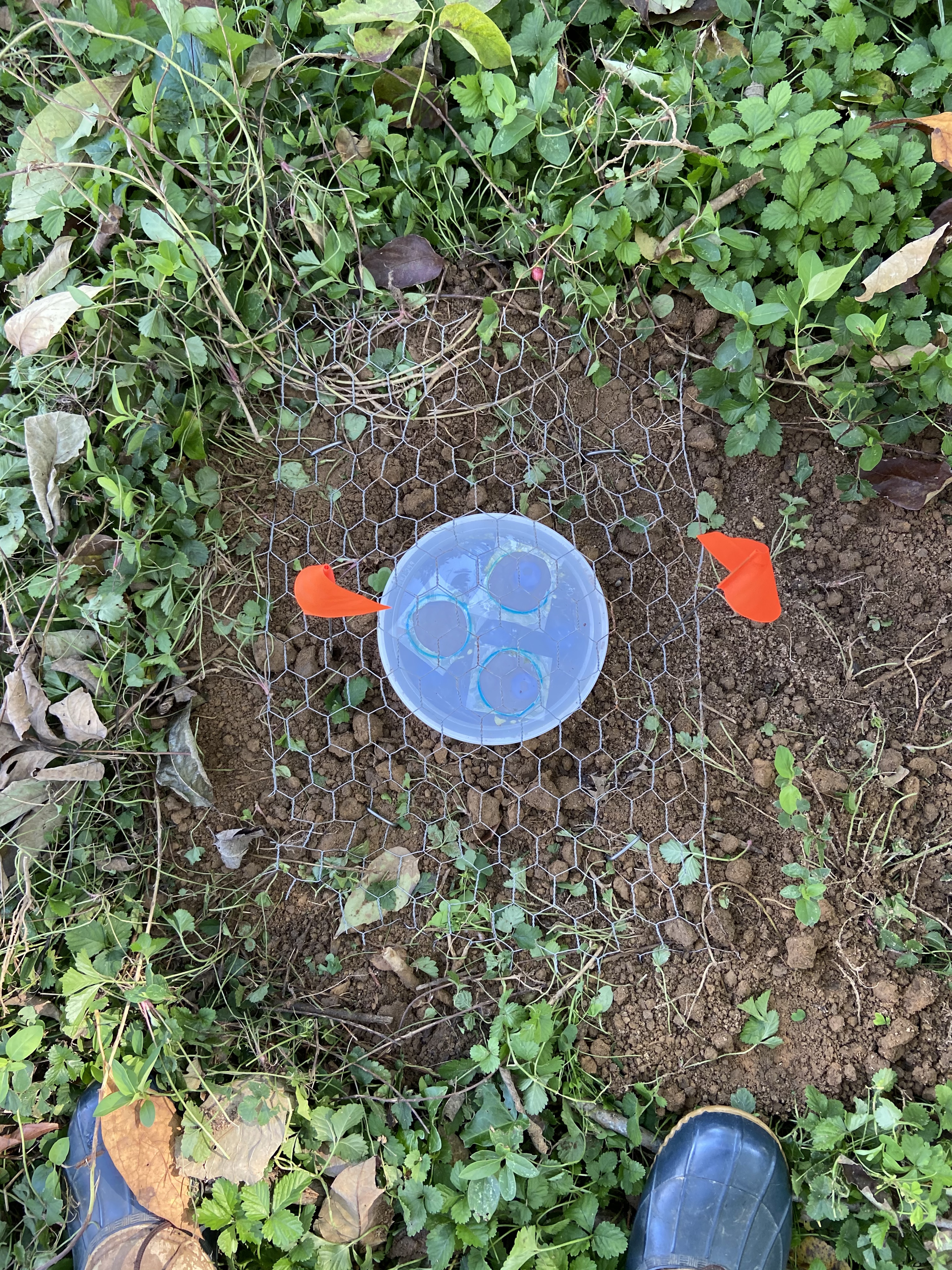

Supplement: Supplementary file 1 [file insects-12-01000-s001.zip › Figure S3-b.jpg]
